# INSPIRE (**IN**vestigating **S**ocial and **P**ract**I**cal suppo**R**ts at the **E**nd of life): Pilot randomised trial of a community social and practical support intervention for adults with life-limiting illness

**DOI:** 10.1186/s12904-015-0060-9

**Published:** 2015-11-24

**Authors:** Kathleen McLoughlin, Jim Rhatigan, Sinead McGilloway, Allan Kellehear, Michael Lucey, Feargal Twomey, Marian Conroy, Emillio Herrera-Molina, Suresh Kumar, Mairead Furlong, Joanne Callinan, Max Watson, David Currow, Christopher Bailey

**Affiliations:** Department of Psychology, Maynooth University, Maynooth, Co., Kildare, Ireland; Milford Care Centre, Limerick, Ireland; Faculty of Health Studies, University of Bradford, Bradford, UK; Newhealth Foundation, Sevilla, Spain; Institute of Palliative Medicine, Kozhikode, Kerala India; Queens University Belfast and Northern Ireland Hospice, Belfast, UK; Flinders University, Adelaide, Australia; University of Southampton, Southampton, UK

**Keywords:** Randomised Controlled Trial, Palliative care needs, Practical support, Social support, Volunteers, Community, Adults, Carers, Quality of life, INSPIRE, Home

## Abstract

**Background:**

For most people, home is the preferred place of care and death. Despite the development of specialist palliative care and primary care models of community based service delivery, people who are dying, and their families/carers, can experience isolation, feel excluded from social circles and distanced from their communities. Loneliness and social isolation can have a detrimental impact on both health and quality of life. Internationally, models of social and practical support at the end of life are gaining momentum as a result of the Compassionate Communities movement. These models have not yet been subjected to rigorous evaluation. The aims of the study described in this protocol are: (1) to evaluate the feasibility, acceptability and potential effectiveness of The Good Neighbour Partnership (GNP), a new volunteer-led model of social and practical care/support for community dwelling adults in Ireland who are living with advanced life-limiting illness; and (2) to pilot the method for a Phase III Randomised Controlled Trial (RCT).

**Design:**

The INSPIRE study will be conducted within the Medical Research Council (MRC) Framework for the Evaluation of Complex Interventions (Phases 0–2) and includes an exploratory two-arm delayed intervention randomised controlled trial. Eighty patients and/or their carers will be randomly allocated to one of two groups: (I) Intervention: GNP in addition to standard care or (II) Control: Standard Care. Recipients of the GNP will be asked for their views on participating in both the study and the intervention. Quantitative and qualitative data will be gathered from both groups over eight weeks through face-to-face interviews which will be conducted before, during and after the intervention. The primary outcome is the effect of the intervention on social and practical need. Secondary outcomes are quality of life, loneliness, social support, social capital, unscheduled health service utilisation, caregiver burden, adverse impacts, and satisfaction with intervention. Volunteers engaged in the GNP will also be assessed in terms of their death anxiety, death self efficacy, self-reported knowledge and confidence with eleven skills considered necessary to be effective GNP volunteers.

**Discussion:**

The INSPIRE study addresses an important knowledge gap, providing evidence on the efficacy, utility and acceptability of a unique model of social and practical support for people living at home, with advanced life-limiting illness. The findings will be important in informing the development (and evaluation) of similar service models and policy elsewhere both nationally and internationally.

**Trial registration:**

ISRCTN18400594 18^th^ February 2015.

## Background

Most people want to be cared for and to die at home [1] and in most developed countries, this choice is supported by public policy. For example, in Ireland national policy has, for more than 50 years, focused on trying to ensure that older people are enabled to live in their own homes or to ‘age in place’ for as long as possible, with the help of both formal and informal services [2]. However, despite the development of specialist palliative care hospice at home services and models of primary care, people who are dying, and their families, can experience great isolation and can feel excluded from social circles and distanced from their communities [3]. A recent review published by the Social Care Institute for Excellence [4] highlights the detrimental impact of such loneliness and social isolation on health and wellbeing, and, arguably this may impact most on the quality of life of people with palliative care needs and their families [5]. Social isolation can also pose a barrier to the successful execution of instrumental activities of daily living. For example a study by Macmillan in the United Kingdom (UK) reports that more than 1 in 6 (18 %) people living with cancer were unable to collect a prescription for their medication, whilst this proportion increased to 1 in 4 (24 %) amongst women [6]. This is only one example of the many small practical, but significant needs of those who find themselves socially isolated due to end stage illness.

Existing evidence suggests that informal support networks may help to naturalise’ dying; offer better support to the person and family; reduce isolation; target professional support more effectively and equitably; and enable choice to die at home [7]. Such networks are at the heart of Health Promoting Palliative Care theory [8], one component of which, encourages communities to care for people and their families as they encouter death and in turn build Compassionate Communities [9]. These may be described as communities that recognise *“care for one another at times of health crisis, and personal loss is not simply a task solely for health and social services, but is everyone’s responsibility”* [10].

Such community-led interventions focus on the development and delivery of a social/professional model of care and support for people living at home with palliative/end of life care needs and are usually provided and/or led by the community. Thus, they tend to involve the use of volunteers and/or naturally occurring personal/informal networks [11, 12, 13]. The idea underpinning these models is that, by drawing on the resources of the community, it is possible, not only to meet a person’s social and/or practical needs, but also more broadly to build capacity and resilience in the community and naturalise the process of care, dying, death and bereavement.

Community-led interventions for people with palliative care needs and their carers have been implemented in several countries internationally and initial positive findings/outcomes reported [11]. For example, in the UK, Severn Hospice has developed a Compassionate Communities (Co-Co) befriending model where volunteers provide practical help to people facing the end of life with day-to-day activities such as shopping, gardening and the collection of prescribed medication. The available evidence suggests that this model has reduced patient isolation and led to fewer unscheduled healthcare visits to primary care and other allied health services, thereby reducing demand on health service staff and budgets [12].

In India, the Neighbourhood Network in Palliative Care (NNPC) involves the community in providing social, spiritual and emotional support to people at home, facing the end of life, supporting more than 2500 patients per week [13–15]. In Australia, the Home Hospice Volunteer Mentoring Model (now known as LifeCircle) works alongside medical and other essential home based services providing support for carers, helping them to gather a support team and avoid burn-out. In Spain, the SAIATU project enables the provision of home-based social support services to complement palliative clinical services, and to enhance the care provided to individuals living with advanced illness and their families. An evaluation showed that those who had received this intervention had fewer unscheduled health service visits (Accident and Emergency, Out Patient Department and hospital admission) when compared to the control group. In addition, both patients and families rated the intervention positively, whilst the cost effectiveness of the model was also demonstrated [16, 17].

Internationally, there is a growing policy impetus toward the increased provision of community-led interventions as described above. However, the development of models of social and practical care and support as described above is relatively new, and robust study designs are necessary to determine whether models of social and practical care and support are effective. Conducting randomised controlled trials with people facing the end of life can be both ethically and methodologically challenging and therefore it is important to progress methodically through the phases of Medical Research Council (MRC) Framework for Complex Interventions [18, 19], examining both the acceptability and feasibility of the intervention and the associated study design [20].

### Aim and objectives

The overall aims of the INSPIRE study are to (1) develop a greater understanding of the practical and social needs of people living with advanced life limiting illness and (2) assess the feasibility, acceptability and subsequent effectiveness of The Good Neighbour Partnership (GNP), a volunteer-led model of social and practical care/support for community dwelling adults living with advanced life-limiting illness in Limerick, Ireland.

These aims will be achieved by working through the first three phases of the the MRC Framework for the Evaluation of Complex Interventons [18, 19]. Within Phase II of the Framework a pilot delayed intervention randomised controlled trial will be conducted to assess both the effectiveness of the GNP and associated research methods, in advance of scaling up to a larger Phase III RCT.

The specific objectives of the delayed intervention randomised controlled trial are to determine whether the GNP can:

1. Reduce unmet social and practical need;
2. Reduce unplanned health service utilisation;
3. Improve overall quality of life;
4. Reduce loneliness;
5. Increase social capital;
6. Improve social support;
7. Alleviate caregiver burden.

The research team will also seek to:

- Develop, implement and evaluate a brief training programme delivered to Compassionate Communities Volunteers prior to the implementation of the GNP;
- Assess the impact of participation on volunteers’ death anxiety, death self-efficacy and self-reported knowledge and confidence regarding eleven skills considered important for volunteers to successfully deliver the intervention; and
- Conduct a process evaluation, examining the feasibility and acceptability of both the model of care and method of evaluation from all perspectives (patient, carer, health professional and volunteer).

## Methods

### Participants and settings

The care of community dwelling adults with advanced life-limiting illness in Limerick is organised in line with the person’s needs, through the relevant Health Service Executive (HSE) Primary Care Team and where necessary via Milford Care Centre, a Specialist Palliative Care provider. Limerick City and County have a combined population of 191,809, half of whom (95,894) live in Limerick City [21]. Whilst there are significant clusters of the population residing around large towns, there are a large number of people living in isolated rural locations. It is anticipated that there will be a 25:75 % urban/rural mix in this study, based on an analysis of death notice data comprising 1650 deaths in the last 12 months.

### Design

**Fig. 1:**
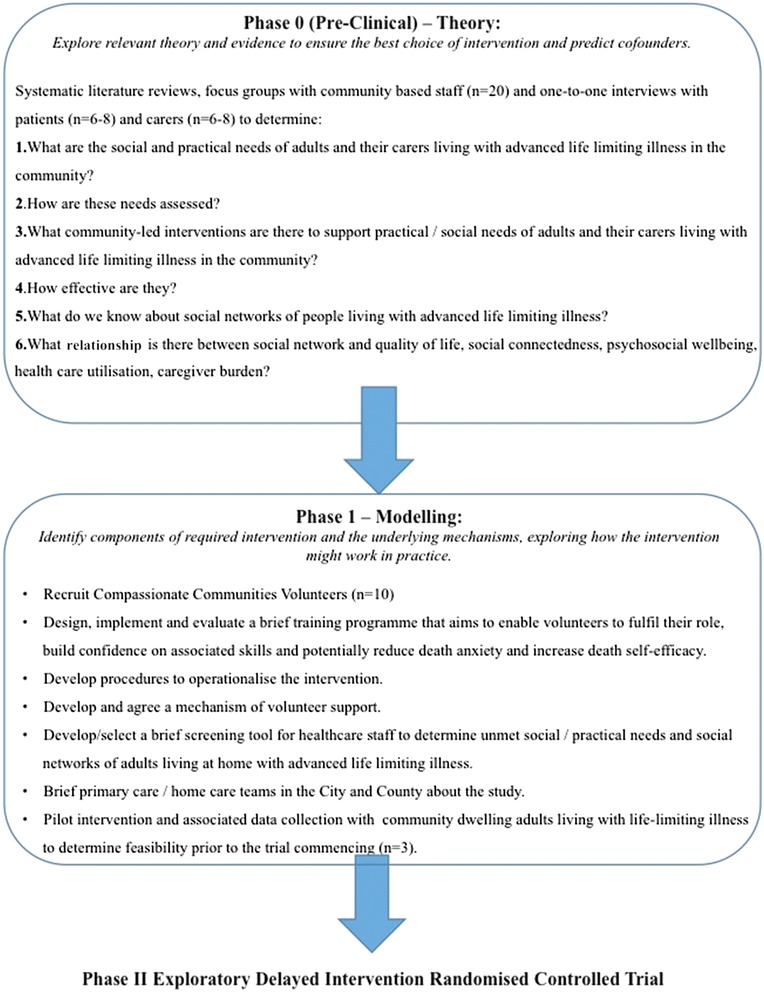
Overview of Phase 0-1 of the INSPIRE Study

**Fig. 2:**
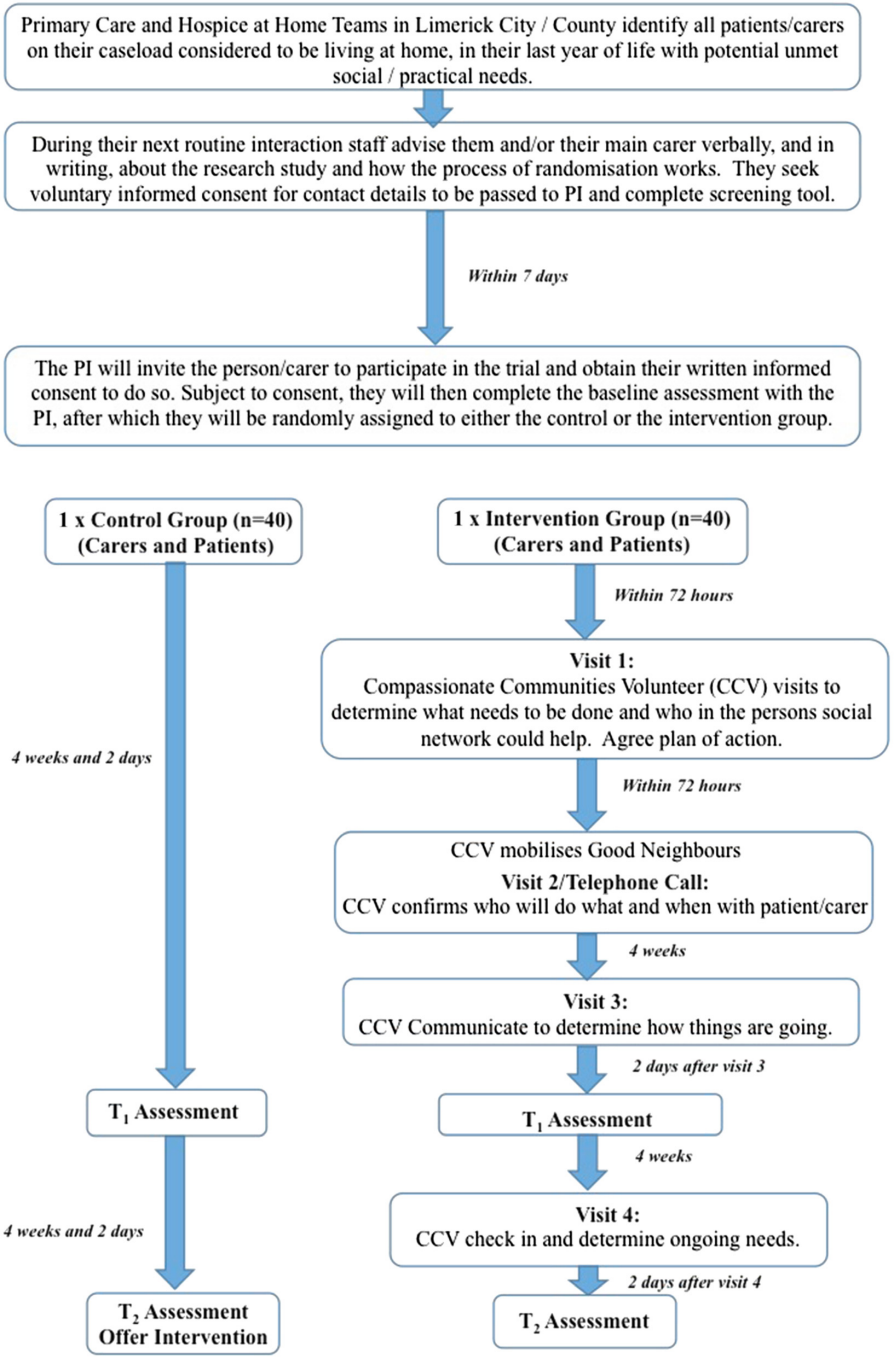
Overview of Phase 2 of the INSPIRE Study

The evaluation will be guided by the MRC Framework for the Evaluation of Complex Interventions [18, 19]. In the INSPIRE study, the first three phases of the Framework will be completed (i.e., from pre-clinical phases to phase II – an exploratory trial) as outlined below and in Figs. 1 and 2.

### Phase 0 (pre-clinical) – Theory: Explore relevant theory and evidence to ensure the best choice of intervention and predict cofounders

A significant body of work has already been completed by KMcL and JR to design the Good Neighbour Partnership intervention. The study has been under discussion and development for over two years. Much of this work is undocumented and therefore this study will satisfy Phase 0 of the MRC Framework of Complex Interventions, by documenting a theoretical basis to this study. This will include:

(1) A scoping review to determine what is known from published evidence about the social and practical needs of adults living with advanced life limiting illness. (2) A systematic review of community-led practical and/or social support interventions for adults living at home with palliative and end of life care needs. (3) A small number of interviews conducted with: (a) community dwelling adults living with advanced life-limiting illness in Limerick (n ≈ 6–8); (b) carers of community dwelling adults living with advanced life-limiting illness in Limerick (n ≈ 6–8); and (c) two focus groups with the primary care team/Hospice at Home teams to determine the most important practical and social concerns for people living with advanced life limiting illness, the needs of carers, the assessment tools in use and the problems faced by staff.

The results from this work will inform the final model of intervention for the trial.

### Phase I – Moldelling: Identify components of required intervention and the underlying mechanisms, exploring how the intervention might work in practice

The intervention requires a number of components to be in place prior to commencment, including the recruitment and training of at least 10 Compassionate Communities Volunteers to deliver the intervention and the development of associated procedures to operationalise the intervention (e.g. forms and procedures for handling money/medication etc.). A mechanism of volunteer support must also be developed. This phase includes the design, implementation and evaluation of a brief training programme designed to enable volunteers to fulfil their role, build confidence in communication and other skills associated with the Good Neighbour Partnership and potentially reduce death anxiety and increase death self-efficacy.

A brief screening tool for healthcare staff will be selected, or, if necessary, developed and tested, based on the findings from Phase 0; this will be used in Phase II to screen people in the community for their eligibility to enter the trial. A tool to explore unmet social/practical needs and social networks of adults living at home with advanced life limiting illness and/or their carers, will also be sourced, or if necessary developed.

Briefing sessions regarding the study will be held with all primary care/home care teams in the City and County through existing mechanisms of communication with these groups.

The intervention and associated data collection tools will be piloted with three community dwelling adults living with life-limiting illness to determine feasibility prior to the trial commencing (Fig. 1).

### Phase II – exploratory delayed intervention randomised controlled trial

Based on the outcome of Phases 0 and I, an exploratory delayed intervention randomised controlled trial (RCT) will be undertaken to assess the overall effectiveness of the GNP. Ethically, a delayed intervention is considered appropriate since it is not known at this stage, whether or not the intervention is effective. People in the intervention arm will receive the intervention immediately following screening, whilst those in the delayed intervention/control group will receive their usual care, and can then elect to receive the intervention eight weeks after screening. The RCT will be conducted with reference to the CONSORT statement [22].

### Randomisation

Randomisation will be conducted independently (and after the baseline interview) by a statistician, using the minimisation method [23]. This method will yield an equal balance between groups by level of assessed social and/or practical need and social network. This method ensures a balanced distribution of potentially prognostic factors, even in small trials [24]. The control group will be monitored for contamination by the intervention at the eight week interview where a question will be included asking participants in the control group if they have had contact with anyone who have received or volunteered as part of the intervention.

### Recruitment, consent and baseline interviews

Public Health Nurses (PHNs) and the Hospice at Home Team staff will identify all patients/carers on their caseload considered to be living at home, in their last year of life with potential unmet social/practical needs who meet the eligibility criteria for the study. During their next routine interaction, staff will advise the person and/or their main carer verbally, and in writing, about the research and how the process of randomisation works. They will then seek their voluntary informed consent for their details to be provided to the Principal Investigator (PI) (KMcL). The PI will visit the person within one week to invite them to participate in the trial and to obtain their written informed consent to do so. Subject to consent, they will complete the baseline assessment of social and practical need and social network with the PI, together with a battery of research measures (see below), after which they will be randomly assigned to either the control or the intervention group.

### Sample size

It is anticipated that up to 80 people will be recruited to the INSPIRE trial. The trial is intended as a pilot, to test procedures for acceptability, estimate the likely rates of recruitment and retention of the participants, and permit calculation of appropriate sample sizes based on the primary outcome measure for future larger scale studies. Given the exploratory nature of the trial, we estimate that the proposed sample size of 80 is a realistic estimate based on the nature of the intervention and the possible size of the study population, as well as what is feasible within the one year timeframe for the nature of the Phase II element under the MRC Framework. A sample of 80 is also larger than that used in a similar study using a delayed intervention RCT design to determine the effectiveness of a new palliative care service [25]. Furthermore, as the precise choice of outcome measure depends on the findings from Phase 0 of the study, it is not possible at this stage to conduct a power analysis. However, when we have identified the primary outcome measure, we will be in a better position to estimate sample size based on the expected difference on the outcome measure between the control and intervention group, from use of the measure with a similar population. This study has three units of analysis: (1) the person living with life limiting illness; (2) the carer and (3) the volunteers.

### Inclusion criteria

Community dwelling adults (over 18 years) living with a life-limiting illness in Limerick, considered by a member of the primary care/hospice at home team to be in their last year of life and/or their carer are eligible to participate in this study. Participants must have an advanced diagnosis of one of the following conditions: Cancer, dementia, frailty, neurological disease, heart disease, vascular disease, respiratory disease, kidney disease or liver disease, and at least two of the general indicators of deteriorating health as outlined in the Supportive & Palliative Care Indicators Tool (SPICT^TM^) [26]. In addition, the person must have: (a) unmet social and/or practical needs; or (b) be socially isolated or (c) rely on just one other person to meet their needs. Those who meet these criteria, as outlined on the brief screening tool are eligible for inclusion in the RCT.

### Exclusion criteria

In the event that a person with a life limiting illness is not able to engage in the study (as indicated by the healthcare professional responsible for their care), due to their condition or a cognitive impairment, data will not be collected from them directly; instead, their carer will be given the option to engage and complete measures relevant to them.

Children and young people under the age of 18 years are excluded from this study.

### Services as usual (control)

People living in the community with advanced life limiting illness receive a variety of services provided mainly by the HSE Primary Care Team and, in the event of having specialist palliative care needs, are offered multidisciplinary specialist palliative care services via Milford Care Centre’s Hospice at Home team. Depending on their needs, they may also avail of acute hospital admission, outpatient clinic or hospice admission. Non-Governmental Organisations (e.g. the Carers Association and Alzheimer Society Ireland) provide information on services and deliver support groups. In addition, they may be supported by natural networks of carers, family members and friends and the wider community. Participants in the control group will complete measures at baseline, four weeks and eight weeks as outlined below. At eight weeks, they will be offered the opportunity to engage in the Good Neighbour Partnership for an eight week cycle of intervention. This will then be reviewed.

### The intervention: the Good Neighbour Partnership (GNP)

The Good Neighbour Partnership can assist the person affected by advanced illness, and their family, to find the extra social and practical support required from within their community by making links with those living close-by who would like to help. The Partnership can identify and mobilise additional help for activities such as walking the dog, doing the shopping, collecting a prescription, going to the library, filling a coal bucket, lighting the fire, mowing the lawn, making a snack, tidying up or sitting with a person who needs a break. It does not involve providing personal or physical care, heavy lifting of people/objects nor does it provide help with medical or financial matters.

At least 10 Compassionate Communities volunteers will be recruited and trained to facilitate the Good Neighbour Partnership over an 8-week intervention period. The role of a Compassionate Community Volunteer with the Good Neighbour Partnership is to make the link between a person/family living with palliative care needs at home, and those in their circle of community who are able to offer support – to seek out and enlist the “Good Neighbour” capacity within local communities. All volunteers will be expected to demonstrate:

- Maturity, common sense and the ability to be discrete and sensitive
- A good understanding of ethical/confidentiality issues
- The ability to be confident and out-going, relate well to others and communicate effectively
- A respectful and non-judgmental approach at all times
- A good sense of humour
- Good organisational skills and ability to complete paperwork
- A good sense of personal boundaries and a clear understanding of the purpose of the role

Volunteers will be nominated by a community organisation, or by a person of good standing. They will have Garda (Police) Clearance; references will be checked and selection will be by interview with KMcL and JR. They will be provided with initial training and ongoing support by Milford Care Centre. As part of that training, they will be given a manual that has already been developed to help them understand their role, what is expected of them and what they can expect from Milford Care Centre. Insurance will be provided by Milford Care Centre and has already been agreed with the insurer. The volunteer recruitment and selection process has already commenced and fourteen volunteers have been selected for training.

The Good Neighbour Partnership Co-ordinator will assign a Compassionate Communities Volunteer, taking into account the profile of the person requiring support, their age and gender, geographical location, personality and the volunteer’s availability and experience. It is anticipated that Compassionate Communities Volunteers will meet with the person up to four times during the 8 week cycle.

The new intervention will be offered in addition to the services as usual outlined above. This is designed to complement existing services and not to duplicate or replace them. The intervention will be informed by Phase 0/I of this study, but an outline of what is expected, is provided in Fig. 2.

#### Visit one

Within 72 h of initial screening and allocation to the intervention, the assigned Compassionate Communities Volunteer visits the person at a mutually agreed time, in the person’s own home, to identify their social and practical needs and the type of support required. They identify with the person, who in their circle of community they would be happy to approach, to enable these needs to be met. We refer to these people as “Good Neighbours”.

An agreement will be reached regarding a plan of action. This may involve the person requiring support directly approaching the identified Good Neighbours to enable their needs to be met, perhaps agreeing on a formula of words to “break the ice”. Alternatively, it may also involve the Compassionate Communities Volunteer directly asking the agreed Good Neighbours to engage in the tasks identified. In the event that no-one has been identified in the person’s circle of community, then an agreement will be reached to approach community organisations and/or Milford Care Centre’s bank of Compassionate Communities Good Neighbours to determine if they are in a position to enable the need to be met.

#### Visit two/or phone call

Once agreement has been reached regarding who will complete the specific tasks, the Compassionate Communities Volunteer will report back to the person requiring support, to update them as to who will do what, and when. It is anticipated that this visit will take 20 min and in some cases, a phone call may suffice. Depending on the situation, it may be necessary for the Compassionate Communities Volunteer to accompany the Good Neighbours completing the task on their first visit, to introduce them to the person who requires support. Assistance is provided without an expectation or implication of payment or other reward or benefit.

#### Visit three

Four weeks after the first visit, the Compassionate Communities Volunteer will visit again, to determine if the new arrangements/systems are working well, or if there needs to be any changes to the plan/modified supports. A mid-way interview will also be conducted two days later by the PI. It is anticipated that this visit will take 30 minutes.

#### Visit four

Eight weeks after the first visit, the Compassionate Communities Volunteer will visit again to evaluate the process and determine if any additional support is required. A second eight week cyle of intervention may be offered at this point depending on needs identified, and this may be included as a follow up of the main study. It is anticipated that this visit will take 30 minutes and a final interview will be conducted two days later by the PI.

All visits will be agreed in advance and will be made by appointment only. Compassionate

Communities Volunteers will be asked to keep a record of their visits on a *Good Neighbour Partnership Report Form.* This will be used to record information on date and duration of visits, and types of activity undertaken. Volunteers will be asked to remind the “Good Neighbours” to keep a note of their visits on a separate similar form. At the end of the 8-week cycle, these forms will be returned to the Good Neighbour Partnership Co-ordinator.

Where a participant leaves their home during the eight week cycle (e.g., admitted to hospital) they will continue to receive the intervention and participate in the study if they are willing to do so.

### The trial: data collection and outcome measures

Data will be collected for both groups on a face-to-face basis in the person’s home. This will include standardised questionnaires used to record demographic information, relevant clinical data, social and practical needs and the nature and extent of social networks. Relevant outcome measures will be identified following Phase 0, but some of the kinds of measures that are likely to be used are outlined below.

People living with illness may be asked to complete (with help) measures such as: (i) The FACIT-PAL-14 [27]; (ii) The UCLA 3-item loneliness scale [28]; (iii) The eight-item modified Medical Outcomes Study Social Support Survey (mMOS-SS) [29]; (iv) Items regarding community and generalised trust, cohesion and social inclusion from Lewis’s Social Capital Questionnaire [30] and a measure of social and/or practical need and social network. Carers engaged in the study may complete measures such as: (i) the 11-item Duke Social Support Index [31]; and (ii) the American Medical Association Carer Self-Support Survey [32].

Measures will be completed at T0 (baseline), T1 (4 weeks) and T2 (8 weeks) as recommended by Greene [33]. The place of care over time, use of paid and unpaid resources and unplanned health service utilisation will also be recorded at each time period. The researcher will read out the questionnaires to the person and show potential responses on large print, laminated A4 flash cards to aid those with any visual impairment.

### Process evaluation

A ‘mini’ process evaluation will also be nested within the INSPIRE trial and conducted at the end of the study, when a small, but diverse, number of patients, carer and volunteers will be invited to take part in a one-to-one interview to explore their experience and views of the new intervention and the process of its implementation. In addition, the time taken to obtain informed consent and collect data, exclusions, recruitment and drop-out rates (patients, their family and volunteers) and missing data will be recorded and reported throughout the course of the RCT.

### Ethical approval

An application for ethical approval was submitted to the Mid-West University Hospitals Scientific Research Ethics Committee in September 2014 and was approved in November 2014.

### Data analysis

#### Phase 0

Qualitative data from interviews and focus groups will be transcribed, anonymised and entered into MAXQDA for standard thematic analysis. Other qualitative analytical techniques (e.g., grounded theory) will also be considered.

#### Phase I: implementation and pilot testing of compassionate communities volunteer training

Descriptive and inferential statistics with specific pre- and post- training comparisons and utilising appropriate tests to assess any change over time in death anxiety and confidence in communication.

#### Phase 2: exploratory (parallel group) RCT

Trial data will be cleaned, checked for coding errors and entered into SPSS for analysis. Differences between the control and intervention groups at baseline will be examined. Given that studies, such as that proposed here, can be subject to attrition and missing data [33], we will endeavour to utilise the recommendation from MOREcare [34] to manage these issues should they arise in this study (e.g. examining patterns of missing data and utilising a taxonomy of attrition). The primary endpoint will be change in unmet social and/or practical needs in the intention-to-treat sample. Secondary efficacy endpoints have also been described. Other end-points include death and the person requesting withdrawal from the study.

It is likely that an analysis of covariance (ANCOVA) for the primary endpoint and for secondary endpoints will be conducted, supplemented by a repeated measures analysis. However, at this stage, given the lack of certainty around the total sample size (and its distribution), it is perhaps appropriate to highlight that the method of analysis will involve fitting between group, repeated measures models with two levels of treatment variable (intervention group, control group) and three time points (T0, T1, T2) to the primary and secondary outcome measures. Model assumptions will be checked and where there is evidence of non-normal distribution, outcomes will be transformed, for example by dichotomising or categorising data. Auxiliary analyses will assess the interaction of potential modifiers (for e.g., age, sex, size of social network at baseline) with treatment in the models.

#### Phase 2: experience and impact of engaging as a volunteer

Descriptive and inferential statistics with specific pre- and post–intervention comparisons using appropriate statistical tests (sample size dependent) to determine any changes in the community volunteers with regard to death self-efficacy (as measured by The Death Self-Efficacy Scale [35], fear of death (as measured by The Revised Collett-Lester Fear of Death and Dying Scale [36] and self-reported confidence with the eleven skills considered important for their role.

### Process evaluation

All qualitative data will be transcribed, anonymised and entered into MAXQDA in preparation for analysis. A number of qualitative analytical techniques will be considered, such as grounded theory or Framework analysis.

## Discussion

It is anticipated that the findings from the various elements of the INSPIRE study will represent an invaluable addition to international literature and provide important insights into the effectiveness, efficacy, utility and acceptability of a unique model of social and practical care for people with life-limiting illness. This will help to inform the development of similar models in other jurisdictions and potentially provide the basis for a larger full-scale RCT into the future. If the INSPIRE trial shows that volunteer-led models of social care and practical support are effective, it could provide evidence to show that a relatively low cost intervention can be offered routinely with support from specialist palliative care and primary healthcare providers.
